# Luminescent Cellulose Fibers Modified with Poly((9-Carbazolyl)Methylthiirane)

**DOI:** 10.3390/polym12102296

**Published:** 2020-10-07

**Authors:** Aleksandra Erdman, Piotr Kulpinski, Jadwiga Gabor, Arkadiusz Stanula, Andrzej S. Swinarew

**Affiliations:** 1 Centre of Papermaking and Printing, Lodz University of Technology, Wólczańska 223, 90-924 Łódź, Poland; aleksandra.erdman@p.lodz.pl; 2 Department of Mechanical Engineering, Informatics and Chemistry of Polymer Materials, Lodz University of Technology, Żeromskiego 116, 90-924 Łódź, Poland; piotr.kulpinski@p.lodz.pl; 3 Faculty of Science and Technology, University of Silesia in Katowice, 75 Pułku Piechoty 1A, 41-500 Chorzów, Poland; jadwiga.gabor@us.edu.pl; 4 Institute of Sport Science, The Jerzy Kukuczka Academy of Physical Education, Mikołowska 72A, 40-065 Katowice, Poland; a.stanula@awf.katowice.pl

**Keywords:** lyocell fibers, luminescent fibers, cellulose fibers, security fibers

## Abstract

This article presents the results of research related to the development of cellulose man-made fibers with luminescent properties. The fibers were obtained from regenerated cellulose with the use of the N-Methylmorpholine-N-Oxide (NMMO) method for lyocell (Tencel) fiber formation. The method is named after the cellulose solvent (NMMO) used to obtain the spinning solution. Fibers are formed by the dry–wet spinning method. Due to the characteristic of the lyocell process, the fibers were easily modified to achieve luminescent properties with star-shaped organic compound poly((9-carbazolyl)methylthiirane) (KMT). Fibers were examined on their mechanical parameters with the use of Zwick Z2.5/TN1S tensile testing machine, and the results show the influence of the KMT concentration in the fiber matrix on mechanical parameters of the fibers. The study also attempted to determine the concentration of the modifier in the fibers with the use of UV-VIS Spectrofluorometer JASCO. The luminescent properties of fibers were estimated as well, using Jobin–Yvon spectrofluorometer FLUOROMAX–4, and the results are very promising as the fibers emit blue light in the range of visible light spectrum even for small concentrations of KMT (about 0.1 wt.%).

## 1. Introduction

The technologies of fiber production are developing rapidly along with the progressing global economic growth, the development of many scientific fields and the growing demand for modern textile materials. There is a wide range of nanofibers, optical or bioactive fibers, synthetic or man-made fibers with special properties, or modified natural fibers for special usage [1]. One of the fields that are developing and creating a novel type of fiber is connected with luminescence phenomena and leads to optical or other luminescent fibers’ formation for different, novel application. Miluski et al. [2] a created fiber sensor for temperature measurements with the usage of luminescent rhodamine B agent. Synthetic polymethyl methacrylate (PMMA) fiber was dyed with rhodamine B and coated with silver and polyurethane. The fiber characterized by a high emission of red light was highly sensitive to temperature, and all the advantages could be used for creating composite material monitoring the temperature in the industry. Modification can be carried out in a polymer matrix of fibers, when they are synthetic or man–made fibers. Prahsarn et al. [3] created a novel luminescent substance, the 3,12-Bis(dibutylamine)-7,8-dicyano-5,6,9,10-tetrahydro (5) helicene (M123), and incorporated into polypropylene fiber during the melt–spinning process. The fibers were characterized by different cross-sections and emission of green light under UV excitation. Effective luminescence was achieved with a small amount of modifier M123, which was only 0.05 wt.%. Parola et al. [4,5] carried out research on optical polymer fibers with luminescent properties. The fibers were produced with the extrusion process from poly-(methylmethacrylate) (PMMA) polymer and luminescent dopant, which was incorporated into the reaction chamber. The fibers in the future can find an application as a fluorescent solar concentrator. This kind of material can be produced in bi-component melt spinning as well. Jakubowski et al. [6] obtained luminescent solar concentrator fibers by mixing three thermoplastic polymers: cyclo olefin polymer (COP), polycarbonate (PC) and poly(methyl methacrylate) (PMMA) in the form of granulates and modifying them with luminescent dye Lumogen Red 305. The research shows that fiber-based luminescent solar concentrators (LCS) can be used in photovoltaics. Properly prepared optical fibers can find other applications. Acha et al. [7] obtained fluorescent optical fiber biosensor for the detection of mercury (Hg^2^⁺) ions in aqueous solutions. Active optical fibers can be also obtained by inorganic modification with the use of rare earth ions as a luminescent agent. Zmojda et al. [8] described luminescent properties of active optical fibers doped with lanthanides. They showed that the polymer matrix made of poly(methyl methacrylate) can be relatively easy doped by organometallic complexes of lanthanides (Terbium (III), Europium(III) and Dysprosium(III)).

**Figure 1:**
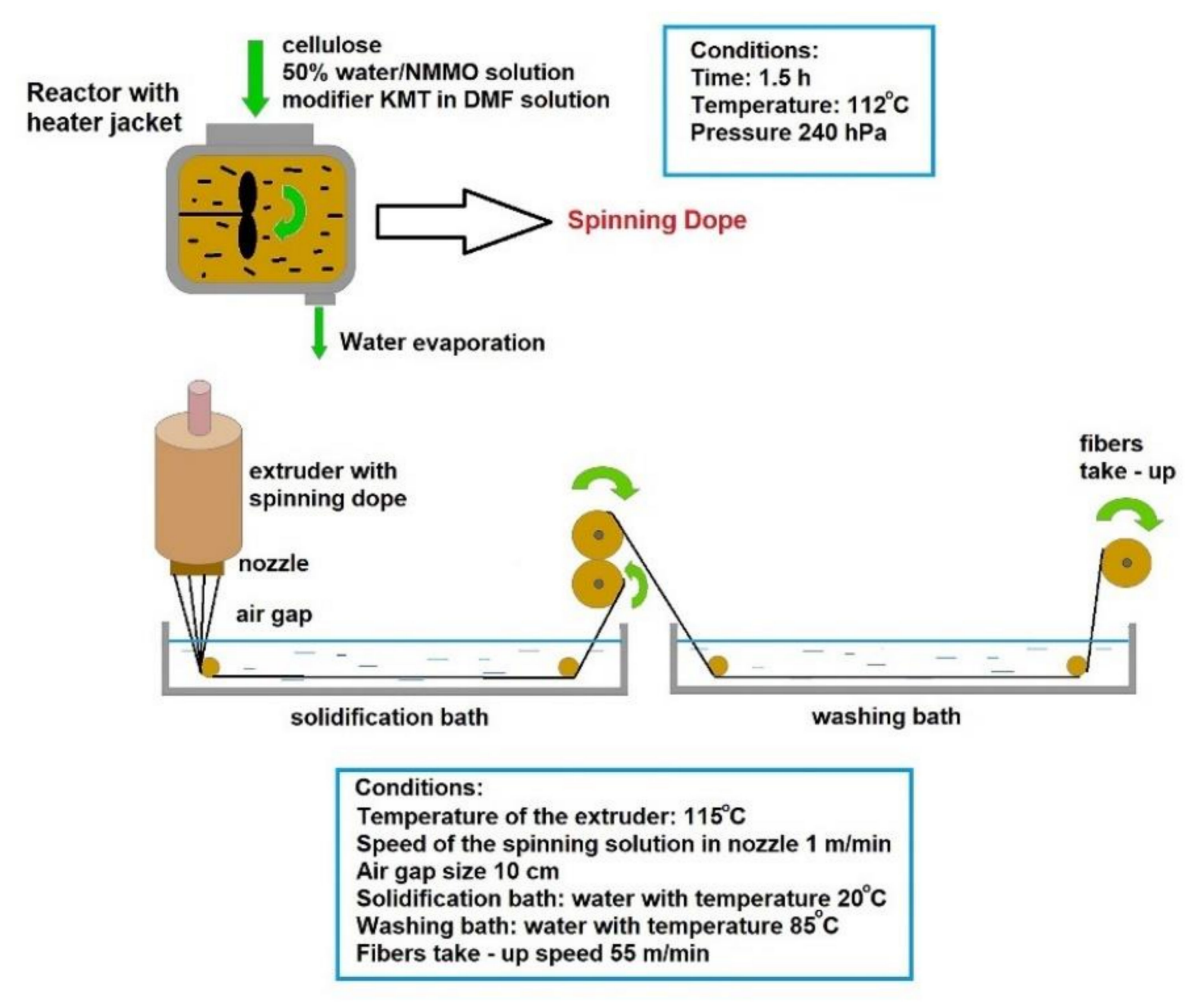
Scheme of man–made cellulose fibers formation in laboratory scale and typical conditions during the process.

The authors of the present paper also have experience in obtaining fibers modified with rare earth ions [9,10,11,12], where the fiber matrix was regenerated cellulose. The fibers were obtained by the N-Methylmorpholine-N-Oxide (NMMO) method [13], using the laboratory equipment and conditions shown on a scheme in Figure 1. The same method was used for luminescent fibers’ formation presented in the article.

Due to the specific characteristic of the fibers’ formation method, there is a wide range of modification possibilities. Using the NMMO method fibers with antibacterial [14,15], magnetic [16], thermochromic [17] or bifunctional magnetic–luminescent [18] properties can be obtained. This method also allows one to produce hybrid fibers, for example, by mixing cellulose and polyacrylonitrile (PAN) materials [19]. Compounds used for fibers’ modification are closed in the polymer matrix, which increases the durability and usability of fibers. According to the research that was previously carried out with inorganic luminescent compounds and the literature research, authors of the presented work focus on creating luminescent man-made cellulose fibers modified with an organic compound, and investigating whether the NMMO method can be used to obtain an effective luminescence of the fibers. The luminescent fibers obtained with this method can find an application as an anti-counterfeiting agent. There is a wide range such fibers that have been developed and used as luminescent additives to the paper products [20,21]. Security fibers can be obtained by melt spinning, diffusion or direct dying [22]. In the case of natural fibers, modification can be achieved by the surface dyeing of the fibers [23].

The main aim of research presented in this paper is to obtain the cellulose fibers with luminescent properties. This goal was achieved by introducing to the polymer matrix an organic luminescent compound—poly((9-carbazolyl)methylthiirane) (KMT). The compound was selected as the fiber modifier due to its properties, which seemed the best due to the method of preparing the fibers and their potential use. KMT is characterized by very good luminescent properties—it is a strong emitter of light. KTM was chosen as a modifier of cellulose fibers also due to its high thermal resistance reaching 285 °C and its relatively high chemical resistance. Due to both mentioned features, the modifier is not degraded under the highly basic conditions and elevated temperature of both spinning dope formation and fiber spinning process. KTM is easily soluble in dimethylformamide (DMF), which greatly facilitates the introduction of this modifier into the fiber matrix. Additionally, organic compounds have advantages over inorganic ones, such as a shorter synthesis time, a larger amount of compound obtained in one process and incorporation to the cellulose pulp in dissolved form, which is simpler and gives very good distribution of modifier in the fiber matrix. In addition, organic modifiers have the advantage that they provide a luminescence effect with small amounts of compound in the fiber matrix, even of point zero—zero of a percent. The fibers are made from the same material as paper (cellulose), and they can be the perfect solution for the protection of documents, security papers, labels, or packages for luxury goods from counterfeit.

## 2. Materials and Methods

Spinning dope was prepared using PLACETAE cellulose pulp (Rayonier, Wildlight, FL, USA) containing 98% wt. of α-cellulose and 50% aqueous solution of N-Methylmorpholine-N-Oxide (NMMO) from Huntsman Holland BV, the Netherlands. For the stabilization of the molecular weight of cellulose in the spinning dope preparation process, propyl ester of gallic acid (Tenox) was used from Aldrich (Gillingham, Dorset, UK). The luminescent modifier of cellulose fibers was photoluminescent star-shaped poly((9-carbazolyl)methylthiirane) (KMT). The modifier was prepared at the Institute of Materials Science, University of Silesia (Katowice, Poland) with the method described in paper Swinarew et al. [24].

To determine the real content of modifier in fibers, the UV-visible spectrophotometry quantitative method was used. Then, the single-point standardization procedure was used to calculate the real content of KMT in fibers.

The concentration of modifier in probes was calculated with the equation:

$$C_{x}=\frac{A_{x}}{A_{s}}C_{s}$$

where:

*C_x_* — Concentration of substance in unknown solution;
*C_s_* — Concentration of standard solution;
*A_x_* — Absorbance of unknown solution;
*A_s_* — Absorbance of standard solution.

Then, the mass of modifier in probes was calculated with the use of the equation:

$$C_{m}=\frac{m_{solute}}{m_{solution}}100$$

where:

*C_m_* — percent by mass.

Finally, the real concentration of KMT in fibers was calculated as follows:

$$C_{KMT}=\frac{measured\;mass\;of\;KMT\;in\;fibers(g)\times \;teoretical\;concentration\;in\;fibers(\%)}{mass\;of\;KMT\;in\;fibers(g)}$$

where:

*C_KMT_* — measured concentration of KMT in fibers.

### 2.1. Instrumentation

Cellulose solutions were prepared using the IKAVISC (Monachium, Germany), laboratory-scale kneader type MKD 0.6-H60.

The mechanical properties of fibers were estimated with a Zwick Z2.5/TN1S tensile testing machine (Ulm, Germany), in accordance with Polish standard PN-EN ISO 5079:1999.

The linear density of fibers was measured according to Polish standard ISO 1973:1995 (E).

Luminescence spectra of the fibers were measured on Jobin–Yvon spectrofluorometer FLUOROMAX-4 (Edison, NJ, USA) with a 150 xenon lamp as an ion source. Spectra were obtained at a temperature of 20 °C and a resolution of 0.1 mm without smoothing and correction.

Estimation of KMT content in modified fibers was prepared with the use of UV-VIS Spectrofluorometer JASCO (Kyoto, Japan). The wavelength of the absorbance peak was 295 nm.

### 2.2. Synthesis of the Modifier

**Scheme 1:**
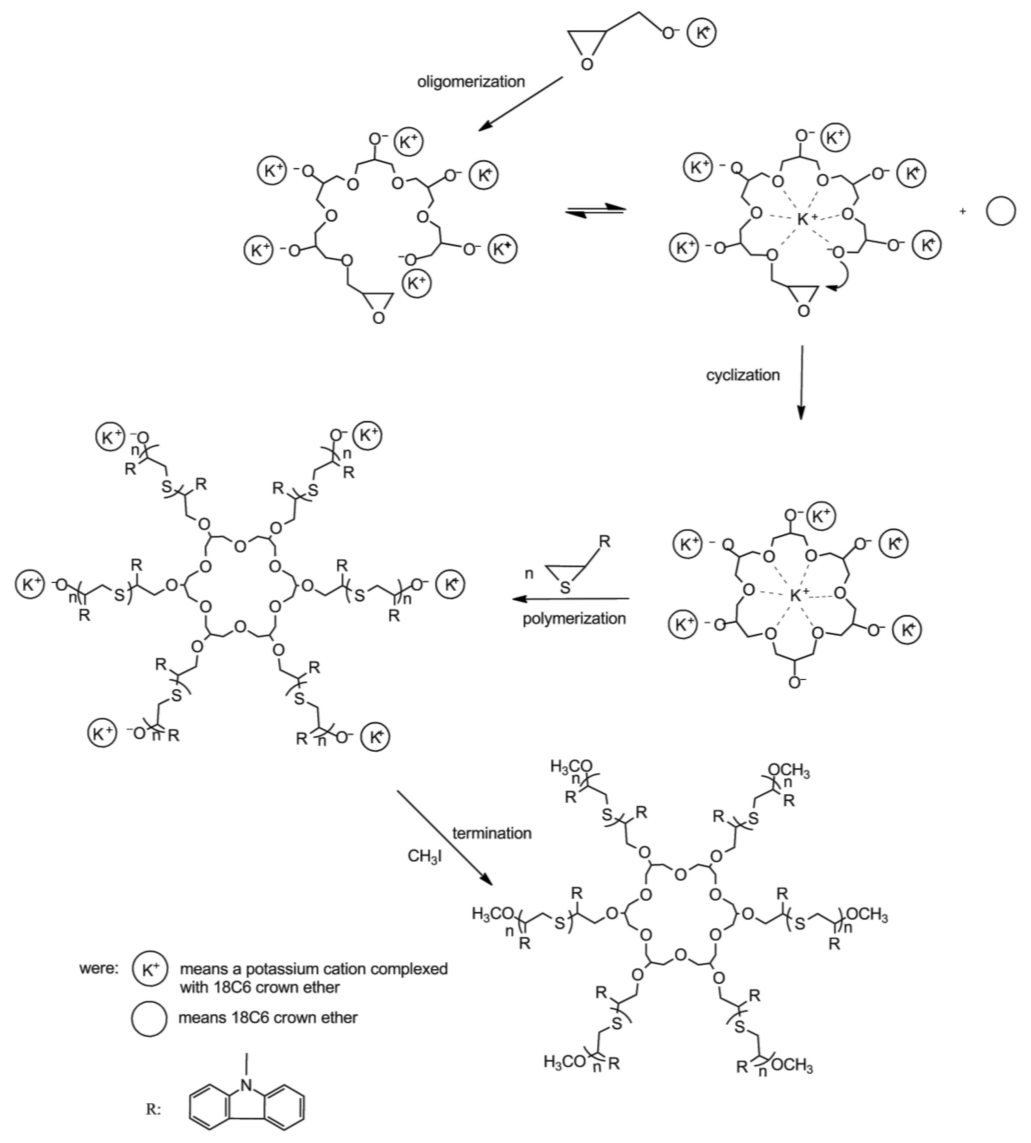
Synthesis of star-shaped luminescent poly((9-carbazolyl)methylthiirane).

The first step of star-shaped luminescent poly((9-carbazolyl)methylthiirane) preparation was monomer formation, where (9-carbazolyl)-methyloxirane was synthesized in the reaction of 9H-carbazole with 2-(chloromethyl)oxirane in the presence of KOH and Na_2_SO_4_ (reagents from Chempur, Piekary Śląskie, Poland). Then, (9-carbazolyl)-methyloxirane was treated with 2,4,6-triochloro-1,3,5-triazine (Sigma-Aldrich, St. Louis, MS, USA) and ammonium thiocyanate (Sigma-Aldrich, St. Louis, MS, USA) in tetrahydrofuran (Avantor Performance Materials Poland, Gliwice, Poland) and monomer (9-carbazolyl)-methytiirane was prepared. The second step was polymerization reaction, where (9-carbazolyl)-methytiirane reacted with oligo(potassium glycidoxide) synthesized in the reaction of glycidol with potassium hydride in the presence of 18-crown-6 in tetrahydrofuran (Scheme 1). The formation of the compound and its luminescent properties are described in detail in the papers [24,25,26].

### 2.3. Preparation of Luminescent Fibers.

The fibers were prepared by a well-known method, used for fiber formation in our previous reports [9,10,11,12]. The difference was in the method of incorporation of the modifier into the spinning dope. In this case, the KMT, which was in the form of white powder, was dissolved in dimethylformamide (DMF). Appropriate amounts of modifier (0.001, 0.01, 0.1, 1 and 10 wt.%) were dissolved in 20 mL of DMF and then added to the cellulose–NMMO–water mixture at the beginning of spinning dope preparation. This method was applied mainly for better dispersion of the modifier in cellulose mass and in the spinning dope as well. DMF was evaporated with water during the cellulose dissolution process. The process of fiber preparation was as follows. Appropriate amounts of cellulose in the form of dry sheets, 50% aqueous solution of N-Methylmorpholine-N-Oxide (NMMO), Tenox, and the modifier KMT were put into the kneader to obtain solutions with 8% of cellulose. The mixture was heated slowly under a low pressure (130 hPa) for about 90 min to get the temperature of 112 °C. The excess amount of water was removed from the solution during the dissolving of cellulose in the NMMO process, until it became homogeneous, transparent, and characterized by good spinning properties. Then, the spinning dope was placed into the chamber of a laboratory-scale piston—spinning device, where the fibers are formed by means of the dry-wet spinning method. The chamber of the spinning device was heated to 115 °C, and the spinning dope was pressed out with a speed of 1 m/min through 18 nozzle holes of 0.4 mm diameter to the air gap of 10 cm long. Extruded polymer ends up in a solidifying bath consisting of water with a temperature of 20 °C, where NMMO was washed out from the fibers and went to another bath with water at a temperature of 85 °C. The fibers were taken up with a speed of 55 m/min and dried at room temperature.

## 3. Results and Discussion

### 3.1. Mechanical Properties of the Fibers

In order to examine the influence of the modifier content in fibers on their linear density, elongation at break and tenacity, series of fibers with different concentrations of KMT were obtained. The results of measurements are presented in Table 1.

The results presented in Table 1 show that there is a dependency between the highest concentration of modifier in fibers and their mechanical properties. However, incorporation of the compound into a fiber matrix of about 1% and less does not influence the linear density, 10% concentration of modifier in fibers increases the linear density by 10% as well, which means that, for high concentrations of modifier in fibers, the fiber mass and diameter are higher. This phenomenon is due to the fact that KMT is a non-fibrous polymer that, at higher concentrations, deteriorates the fiber-forming properties of the spinning solution. A relativity high concentration of modifier makes the spinning solution less susceptible to stretching, with the consequent effect that the fibers have a higher linear mass. The introduction of higher concentrations of the modifier also partially reduces the stability of the formation, which, in consequence, apart from a greater number of filaments brakes, also results in greater dispersions of the linear mass, which is reflected in the statistical data.

The tenacity of the fibers decreases with the increasing of modifier’s concentration in fibers, wherein, for fiber with 10% concentration of luminescent polymer, the drop is very visible and the tenacity is lower by about 45% in comparison to the fiber without modifier. Elongation at break also decreases with the increase in modifier concentration in fibers. Generally, incorporation of larger amounts of organic compounds to the fiber matrix causes the deterioration of the mechanical properties of the fibers. However, the results of the luminescent properties of obtained fibers show that there is no need to incorporate such large amounts of modifier in fibers to obtain good emission of the product.

### 3.2. Luminescent Properties of the Fibers

**Figure 2:**
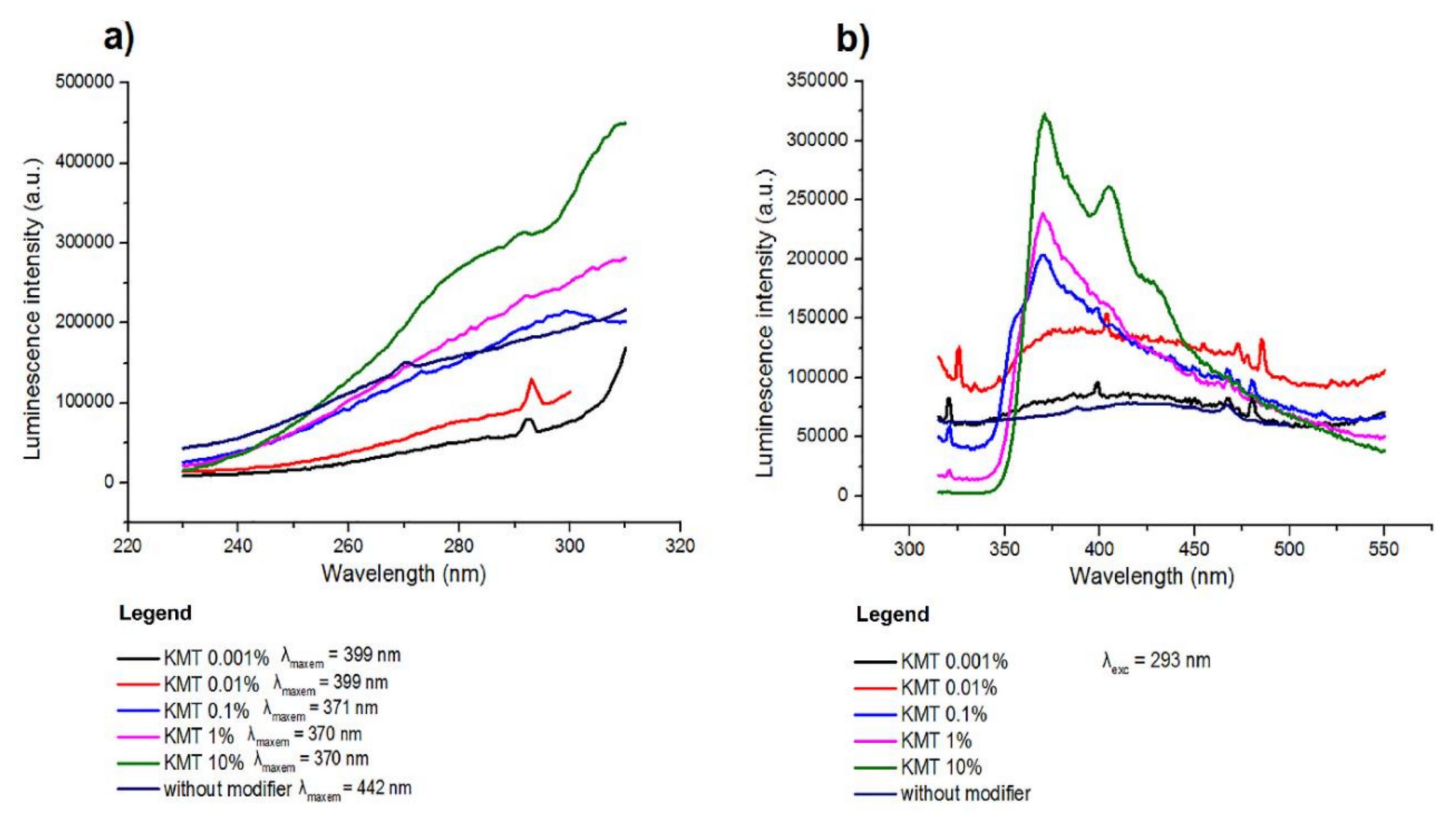
Excitation (**a**) and emission (**b**) spectra of fibers modified with luminescent polymer KMT.

To examine the luminescent properties of the fibers, the excitation and emission spectra were obtained. Fibers modified with different concentrations of KMT were under investigation to study its influence on the luminescent properties of the fibers. The results are presented in Figure 2a (excitation spectra) and Figure 2b (emission spectra).

The results presented in Figure 2 show that the characteristic of curves has changed due to the highest values of the modifier concentration in polymer matrix. This phenomenon can be observed, especially for the emission spectra. The emission of the fibers is characterized by a blue color in the visible light spectrum. There is a sharp peak in emission curves for fibers with 0.1%, 1% and 10% of the modifier, with the maximum at 370 nm wavelength. For fibers with 0.001% and 0.01% of KMT, emission curves have a similar character to the curve for fiber without the modifier, and there is no peak as well. This can be a confirmation for the estimation of the real content of modifier in fibers that, for smaller concentrations (0.001% and 0.01%), there is practically no modifier in fibers. Based on the research results of the luminescent properties of fibers modified with KMT, it can be stated that the optimum concentration of this compound in fibers equals 0.1%. In that case, the optimum emission of light occurs (there is a peak corresponding to emission of light) and the mechanical properties of fibers have not worsened drastically. The incorporation of larger amounts of modifier worsens the mechanical properties of fibers, and, for the lowest concentrations of modifier in fibers, there is no emission of light.

Additionally, the use of low-molecular admixtures to fibers or the creative fiber material, as a rule, significantly reduces the fiber formation and fiber strength and causes deterioration of the luminescent properties. It is most often caused by the agglomeration of the optically active material molecules, which both determines the formation of macroscopic point defects of the polymer chain network, and thus the accumulation of stresses, and the deterioration of the luminescent properties. This quenching occurs as a result of high concentration in the local areas of molecules, which results in the formation of a radiative transition. Such effects are observed for many materials. To avoid the above-mentioned disadvantages, a system with a ring containing sulfur instead of oxygen was used, which resulted in a change of the steric system and eliminating the above-mentioned adverse effects.

### 3.3. Determining the Real Content of Modifier in Fibers

The determining of the real content of modifier in fibers was obtained with the method described in Section 2. Materials and methods and the results are shown in Table 2 and Figure 2.

The results presented in Table 2 show that the concentration of modifier in fibers calculated from UV-VIS measurements differs from theoretical examples. The differences are more drastic for fibers with a lower content of KMT (0.001, 0.01 and 0.1 wt.%). That fact confirms the presumption that the modifier might leak out to the solidification bath during the fiber formation process. For fibers with a higher concentration of modifier in polymer matrix (1 and 10 wt.%), this phenomenon has less impact on KMT concentration in fibers, especially for the one with 10% of modifier in fibers. The results presented above confirm the results of determining the excitation and emission spectra of the KMT fibers (Section 3.2). For fibers with the two lowest theoretical concentrations of KMT (0.01 and 0.001 wt.%), the character of the spectra is similar to fiber without modifier; therefore, the fibers’ luminescence is negligible. The phenomenon of modifier loss during the fiber-forming process is considered by the authors. This problem could be resolved in further research, by, for example, incorporation of the modifier into microcapsules, or the KMT could be chemically bonded with cellulose to prevent the modifier from leaking out to the water during fiber formation. This could improve the durability of fibers and ensure that the theoretically assumed concentration of the modifier will be achieved in the final product.

### 3.4. Optical Observation of Luminescent Fibers and Modified Paper

**Figure 3:**
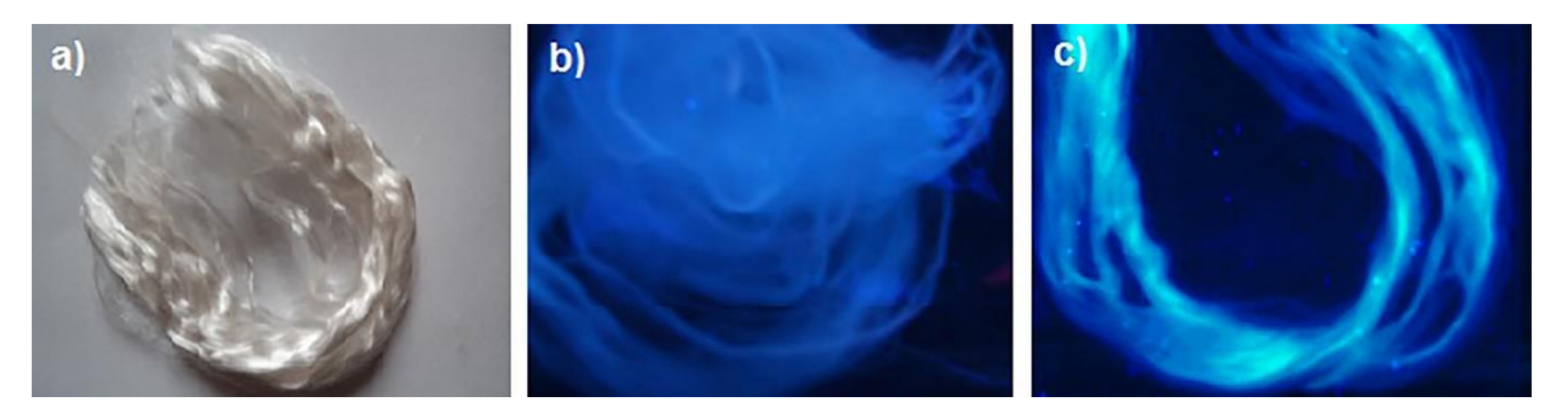
Optical observation of fibers: (**a**) fibers in visible light, (**b**) fibers without modifier under UV light with a 365 nm wavelength, and (**c**) fibers with 0.1 wt.% of KMT under UV with a 365 nm wavelength.

To examine optical properties, the fibers were treated with UV radiation. Figure 3 shows the results.

The results presented above in Figure 3 show that the fibers are characterized by the emission of blue light under the 365 nm wavelength (Figure 3c). The glowing effect does not occur when the fibers without KMT are illuminated (Figure 3b); however, there is a small blue glow that probably comes from the cellulose itself. The fibers were examined with the use of the commercial dual-range lamp (254/365 nm), and the results of the luminescent properties’ examination with a spectrofluorometer (Figure 2) show that, for the fibers obtained in this case, the maximum emission occurs for 370 nm wavelength, so it is likely that the observed effect of glow could be stronger with this wavelength value.

**Figure 4:**
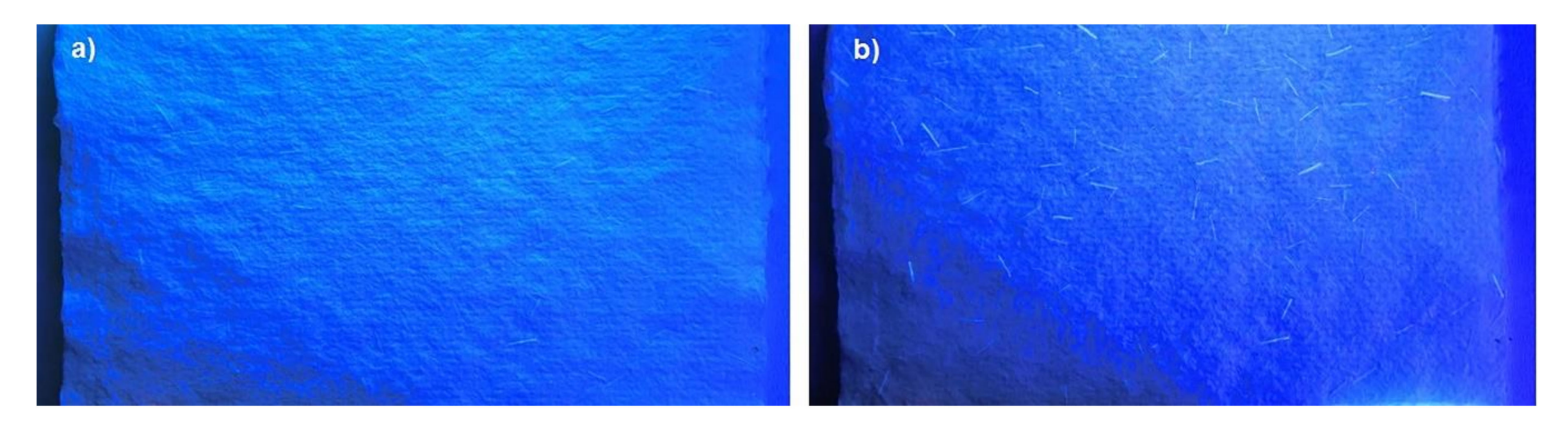
Optical observation of paper modified with fibers containing KMT: (**a**) paper under 254 nm wavelength, no emission of light; (**b**) paper under 365 nm wavelength, visible emission of fibers with KMT.

Due to potential application of obtained luminescent fibers as a securing agent for documents, banknotes, textiles, etc., paper containing them was prepared to examine if they can be visible under certain UV radiation. The paper was prepared as follows. The luminescent fibers were cut into sections 4–5 mm long and then introduced into the vat containing the pulp mixed with water. With the use of a wooden frame with a drainer for manual forming of paper, the mass was taken from the vat. The water was drained from the frame, then the paper was put on felt pads and stamped on a press, then dried at room temperature. Figure 4b shows that the fibers are visible in paper under UV radiation of 365 nm wavelength and that, under a different wavelength (254 nm), the emission of the light of luminescent fibers does not occur.

The paper presented in Figure 4 was prepared with a laboratory handicraft method to observe if the fibers modified with KMT could be visible in the paper mass. The samples of paper were placed under the same UV lamp as the fibers (Figure 3), so the effect of glow is similar. The paper itself has the blue glow independently of the wavelength that was used for the test; however, the luminescent KMT fibers are visible only under the 356 nm wavelength.

**Figure 5:**
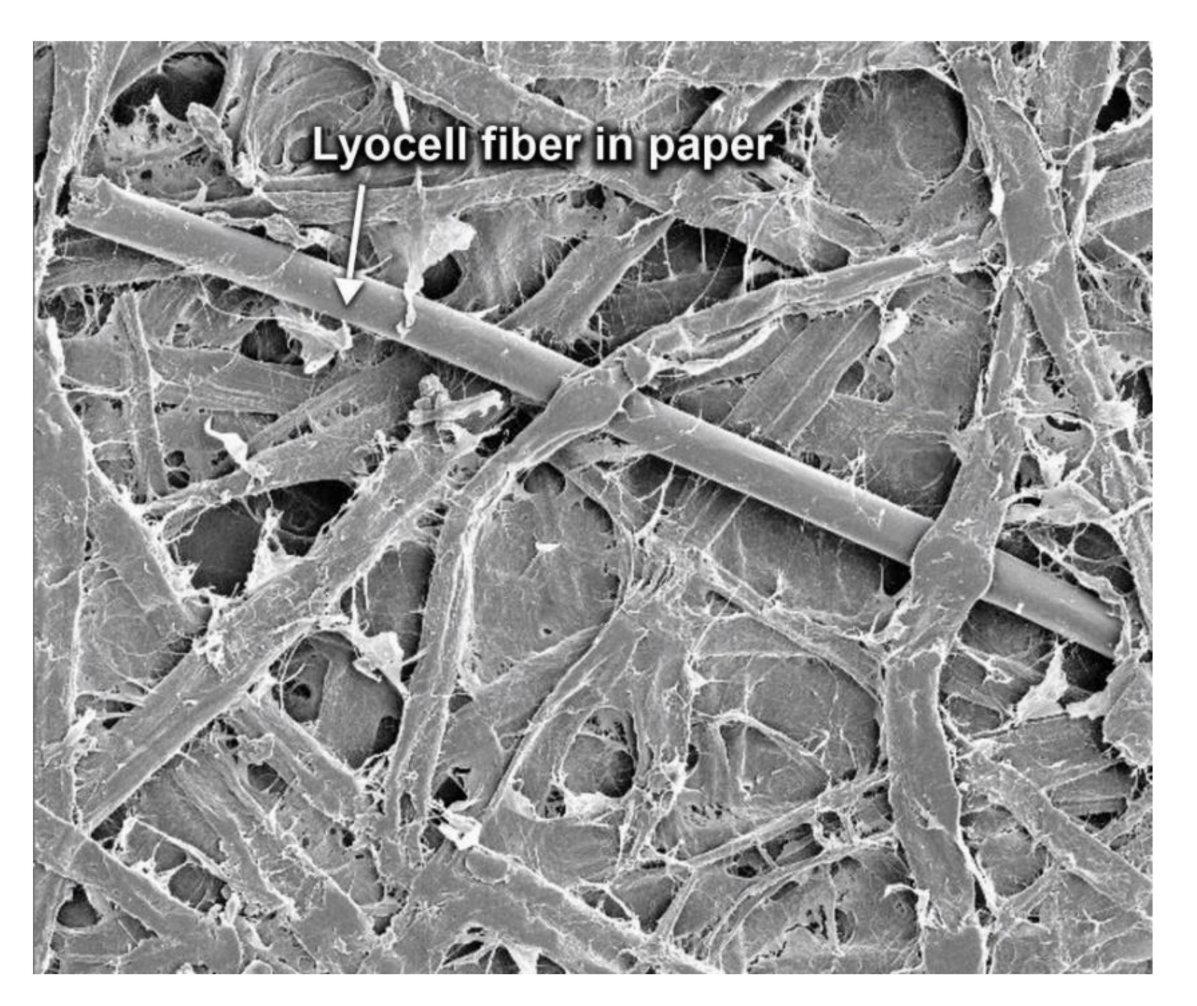
Graphic showing the lyocell fibers in paper structure prepared from Scanning Electron Microscopy (SEM) observation.

For better visualization of the presence of lyocell fibers in paper mass, the graphic with Scanning Electron Microscopy (SEM) observation of luminescent cellulose fiber in the paper was prepared (Figure 5).

As it can be seen on the Figure 5, the man-made cellulose fibers can be easily identified in paper structure, where fibers from paper pulp are flat objects with an irregular surface. The lyocell fibers are characterized by a rounded shape and a smooth surface (Figure 5).

## 4. Conclusions

The cellulose fibers modified with organic luminescent compound (KMT) were successfully prepared by the NMMO method. UV-VIS analyses confirmed the presence of the modifier in the cellulose matrices. The luminescent properties of the modified fibers were verified by spectroscopic analysis. Obtained luminescent fibers are characterized by the blue emission of light and a good optical effect, especially the ones with 0.1%, 1%, and 10% of modifier in the polymer matrix. The optical observation of fibers and paper containing KMT modifier have shown that there is a blue emission under 365 nm excitation.

The results of testing the mechanical properties have shown that a small amount of modifier does not adversely affect the strength of the fibers, only the addition of a 10% modifier significantly reduces their mechanical properties.

To reduce the effect of leaking out of modifier from fibers during the formation process, a solution for better incorporation of modifier in the polymer matrix should be worked out. The fibers obtained in the presented article can be an interesting composite material, especially since the fibers can be easily incorporated into a paper mass, that can be used in protection against counterfeiting of documents and banknotes. Very good mechanical properties allow for the production of both yarns and threads from these fibers and introducing them into various textiles. This technique would protect expensive and high-quality textile products against counterfeiting.

## 5. Patents

There is a polish patent resulting from the work reported in this manuscript:

Swinarew A., Golba S., Flak T., Gabor J., Łężniak M., Kulpiński P. Erdman A., Pęczek B., Modyfikowane włókna na bazie polimerów syntetycznych i/lub naturalnych oraz sposób ich otrzymywania/Modified fibers based on synthetic and/or natural polymers and the method of obtaining them, PL 226783 B1 (2017).

## Figures and Tables

**Table 1:** Mechanical properties of fibers modified with luminescent polymer poly((9-carbazolyl)methylthiirane) (KMT).

No	Concentration of the Modifier (%)	Linear Density (tex)	Standard Deviation for Linear Density	Tenacity (cN/tex)	Standard Deviation for Tenacity Values	Elongation at Break (%)	Standard Deviation for Elongation at Break Values
1	0	0.284	0.0055	30.65	9.27	10.48	1.62
2	0.001	0.303	0.0069	35.96	12.11	8.19	1.08
3	0.01	0.315	0.0140	33.06	11.57	7.37	0.99
4	0.1	0.309	0.0209	26.49	6.71	6.01	0.94
5	1	0.301	0.0161	26.02	7.28	7.46	1.02
6	10	0.343	0.0181	19.56	6.60	5.72	0.96

**Table 2:** Results of the real content of KMT in fiber examination.

Theoretical Concentration of KMT in Fibers (%)	Estimated with the Use of UV-VIS Method Real Concentration of KMT in Fibers (%)
0.001	0.0002
0.01	0.00025
0.1	0.025
1	0.63
10	9.8
